# Separation and Characterization of Phenolamines and Flavonoids from Rape Bee Pollen, and Comparison of Their Antioxidant Activities and Protective Effects Against Oxidative Stress

**DOI:** 10.3390/molecules25061264

**Published:** 2020-03-11

**Authors:** Huifang Zhang, Rui Liu, Qun Lu

**Affiliations:** 1 College of Food Science and Technology, Huazhong Agricultural University, Wuhan 430070, China; huifangzhang@webmail.hzau.edu.cn (H.Z.); liurui@mail.hzau.edu.cn (R.L.); 2 Wuhan Engineering Research Center of Bee Products on Quality and Safety Control, Wuhan 430070, China; 3 Key Laboratory of Environment Correlative Dietology (Huazhong Agricultural University), Ministry of Education, Wuhan 430070, China; 4 Key Laboratory of Urban Agriculture in Central China, Ministry of Agriculture and Rural Affairs, Wuhan 430070, China

**Keywords:** pollen, antioxidant, oxidative stress, hydroxycinnamic acid amides

## Abstract

Phenolamines and flavonoids are two important components in bee pollen. There are many reports on the bioactivity of flavonoids in bee pollen, but few on phenolamines. This study aims to separate and characterize the flavonoids and phenolamines from rape bee pollen, and compare their antioxidant activities and protective effects against oxidative stress. The rape bee pollen was separated to obtain 35% and 50% fractions, which were characterized by HPLC-ESI-QTOF-MS/MS. The results showed that the compounds in 35% fraction were quercetin and kaempferol glycosides, while the compounds in 50% fraction were phenolamines, including di-p-coumaroyl spermidine, p-coumaroyl caffeoyl hydroxyferuloyl spermine, di-p-coumaroyl hydroxyferuloyl spermine, and tri-p-coumaroyl spermidine. The antioxidant activities of phenolamines and flavonoids were evaluated by 2,2-diphenyl-1-picrylhydrazyl (DPPH), 2,2’-azino-bis-3-ethylbenzothiazoline-6-sulphonic acid (ABTS), and ferric reducing antioxidant power (FRAP) assays. It was found that the antioxidant activity of phenolamines was significantly higher than that of flavonoids. Moreover, phenolamines showed better protective effects than flavonoids on HepG2 cells injured by AAPH. Furthermore, phenolamines could significantly reduce the reactive oxygen species (ROS), alanine aminotransferase (ALT) and aspartate aminotransferase (AST) levels, and increase the superoxide dismutase (SOD) and glutathione (GSH) levels. This study lays a foundation for the further understanding of phenolamines in rape bee pollen.

## 1. Introduction

Bee pollen is composed of pollen collected from angiosperm stamens or gymnosperms by worker bees and mixed with a number of enzymes secreted by nectar and bee salivary glands (such as catalase and amylase) [1]. It contains a variety of essential nutrients such as proteins, carbohydrates, lipids, fatty acids, enzymes and vitamins, as well as flavonoids, alkaloids, and phenolamides [2,3,4]. Previous research has confirmed that bee pollen has a variety of nutritional and therapeutic effects, including the prevention of cardiovascular and cerebrovascular diseases, treatment of prostatic inflammation, protection of the liver, prevention of diabetes, and anti-fatigue and skin whitening effects [5,6,7]. Therefore, bee pollen has been widely recognized as a health food [8].

The common characteristic of most chronic diseases is the presence of oxidative stress [9]. Under normal physiological conditions, reactive oxygen species (ROS) can be effectively scavenged by antioxidant defense system. However, when the intrinsic antioxidant system is insufficient to eliminate ROS, oxidative stress will occur, which will cause oxidative injury to lipids, proteins and DNA, leading to cell death and tissue damage [10,11]. Natural antioxidants are considered as effective to scavenge ROS and reduce oxidative stress [12,13,14,15].

Bee pollen has been reported to have good antioxidant activity [16]. Flavonoids and phenolamines are two important components in bee pollen. Flavonoids can scavenge free radicals and ROS, and have protective effects against oxidative stress injury caused by various reasons [17,18]. Phenolamines, also known as hydroxycinnamic acid amides, are polyamide chains coupled with hydroxycinnamic acids [2]. Phenolamines are also a kind of important antioxidants in bee pollen [19], and play a key role in plant development, response to abiotic stress and defense against pathogens and herbivores [20]. However, no study has separated and compared the flavonoids and phenolamines in bee pollen. Moreover, there is no study focusing on the protective effects of phenolamines against oxidative stress in cells.

Rape bee pollen is the highest yield bee pollen in China. Rape bee pollen contains a variety of chemical components, such as proteins, amino acids, lipids, vitamins, minerals, enzymes, polysaccharides, phenolic acids, flavonoids and phenolamines [6,8,21]. Flavonoids in rape bee pollen include flavonoid glycosides and flavonoid aglycones (i.e., quercetin, kaempeferol, isohamnetin), and their antioxidant activities have been reported [8,21]. Phenolamines have also been found in rape bee pollen, but there are few studies on their bioactivity [22].

Therefore, in the present study, we separated and characterized phenolamines and flavonoids from rape bee pollen, and compared their antioxidant activities and protective effects against AAPH-induced oxidative stress in HepG2 cells, aiming to demonstrate the health benefits of phenolamines in rape bee pollen and expand their use in functional food.

## 2. Results

### 2.1. Characterization of Phenolamines and Flavonoids from Rape Bee Pollen

**Figure 1:**
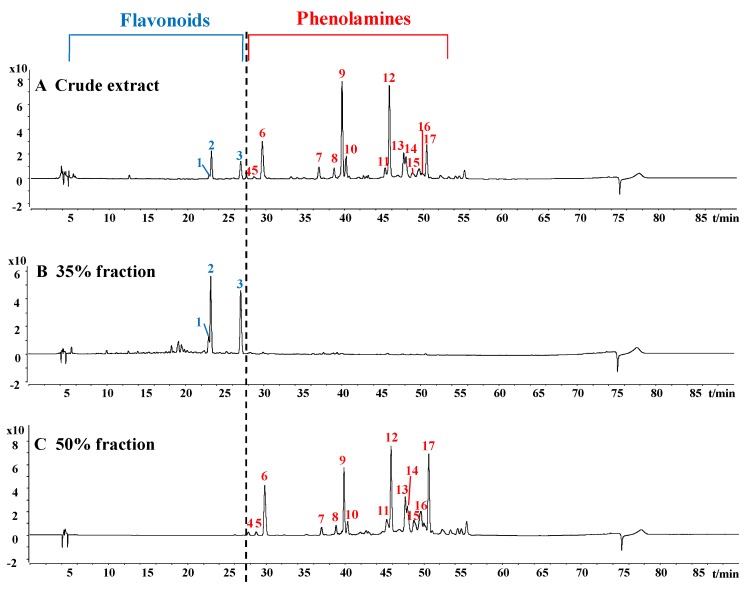
HPLC chromatograms of crude extract (**A**), 35% fraction (**B**) and 50% fraction (**C**) of rape bee pollen (peak numbers correspond to those in Table 1 and Table 2).

The HPLC chromatograms of crude extract and 35% and 50% fractions of rape bee pollen are shown in Figure 1. Through the separation by macroporous resin, the crude extract could be distinctly separated into two parts. The results of HPLC-ESI-QTOF-MS/MS analysis for 35% and 50% fractions are shown in Table 1 and Table 2, respectively.

The proposed formula of peak 1 with [M–H]^−^ ion at m/z 625.1402 was C_27_H_30_O_17_. The two fragment ions of peak 1 were 463.0888 ([M-glc]^−^) and 301.0356 ([M-glc-glc]^−^), indicating that two glucosides are linked to flavonoid aglycones. Based on the fragments and relevant literature [22,23], peak 1 was identified as quercetin di-glucoside. The molecular formula of both peak 2 (m/z 609.1450) and peak 3 (m/z 609.1460) was C_27_H_30_O_16_, indicating that they are isomers. According to a previous report [24], peak 2 and peak 3 were identified as kaempferol di-glucoside. The quercetin di-glucoside has been identified in ragweed pollen and rape pollen [22,23], and the kaempferol di-glucoside has been identified in Ginkgo biloba pollen [24]. The compounds identified in 35% fraction belong to flavonoids.

**Figure 2:**
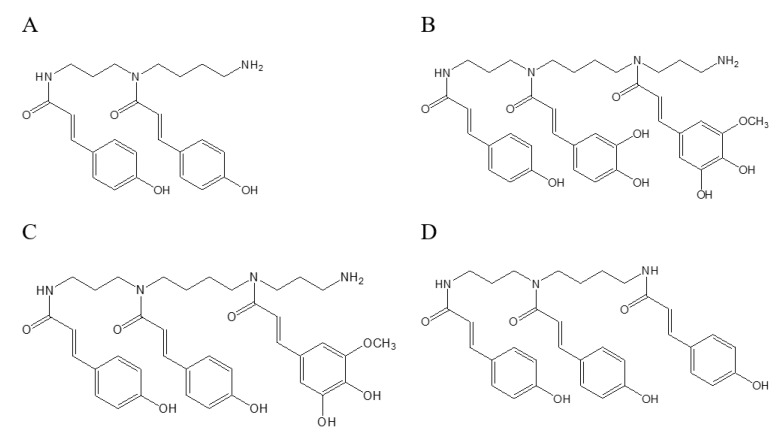
Phenolamines in the 50% fraction of rape bee pollen. (**A**) di-*p*-coumaroyl spermidine, (**B**) *p*-coumaroyl caffeoyl hydroxyferuloyl spermine, (**C**) di-*p*-coumaroyl hydroxyferuloyl spermine, (**D**) tri-*p*-coumaroyl spermidine.

The [M–H]^−^ ions of peaks 4, 5, and 6 had almost identical molecular weights (m/z 436.2247, 436.2241, and 436.2222, respectively), indicating that they are also isomers. Their molecular formula was C_25_H_31_N_3_O_4_, suggesting that they are different from the compounds of the 35% fraction due to the presence of nitrogen. Phenolamines are important components in bee pollen, and contain polyamide chains coupled with hydroxycinnamic acids such as coumaric acid, caffeic acid and ferulic acid [25]. The polyamide chains are mainly spermidine [H_2_N–(CH_2_)_3_–NH–(CH_2_)_4_–NH_2_] and spermine [H_2_N–(CH_2_)_3_–NH–(CH_2_)_4_–NH–(CH_2_)_3_–NH_2_]. [2] Therefore, according to the molecular formula (C_25_H_31_N_3_O_4_), it could be inferred that peaks 4, 5, and 6 were disubstituted hydroxycinnamic acid spermidine, which was further identified as di-p-coumaroyl spermidine based on the fragment information. The two p-coumaroyl residues can be substituted at the N-1 and N-5, N-5 and N-10 or N-1 and N-10 positions of the spermidine chain. Here, one of these structures is shown as an example in Figure 2A. The di-*p*-coumaroyl spermidine has been identified in rape been pollen in previous report [22]. The proposed formula for peak 7 with [M–H]^−^ ion at m/z 701.3173 was C_38_H_46_N_4_O_9_, indicating that it was trisubstituted hydroxycinnamic acid spermine, which was further identified as p-coumaroyl caffeoyl hydroxyferuloyl spermine based on the fragment information. The [M–H]^−^ ions of peaks 8, 9, and 10 were m/z 685.3245, 685.3247, 685.3190, respectively, which were 16 Da lower than that of peak 7 (m/z 701.3173), indicating the replacement of a caffeoyl residue by a coumaroyl residue of p-coumaroyl caffeoyl hydroxyferuloyl spermine. Therefore, peaks 8, 9, and 10 were di-p-coumaroyl hydroxyferuloyl spermine. The trisubstituted hydroxycinnamic acid spermine could be substituted at any three sites of N-1, N-5, N-10 and N-14 in the spermine chain. Here, one of these structures of p-coumaroyl caffeoyl hydroxyferuloyl spermine and di-p-coumaroyl hydroxyferuloyl spermine is shown as an example in Figure 2B,C, respectively. The *p*-coumaroyl caffeoyl hydroxyferuloyl spermine and di-*p*-coumaroyl hydroxyferuloyl spermine were identified for the first time in rape bee pollen in this study. Peaks 11 and 12 need to be further identified. Peaks 13–17 were isomers because they had the same molecular weight (m/z 582), which was 146 Da higher than that of peaks 4, 5, and 6 (m/z 436), indicating that they have one more p-coumaroyl residue. Therefore, peaks 13–17 were tri-p-coumaroyl spermidine, whose structure is shown in Figure 2D. The tri-*p*-coumaroyl spermidine has been identified in ragweed pollen in previous report [23]. The compounds identified in the 50% fraction belong to phenolamines. The results showed that the flavonoids and phenolamines in rape bee pollen were separated by macroporous resin treatment.

### 2.2. Antioxidant Activities of Phenolamines and Flavonoids

To compare the antioxidant capacity of phenolamines and flavonoids from rape bee pollen, three antioxidant assays were employed, including DPPH, ABTS and FRAP.

**Figure 3:**
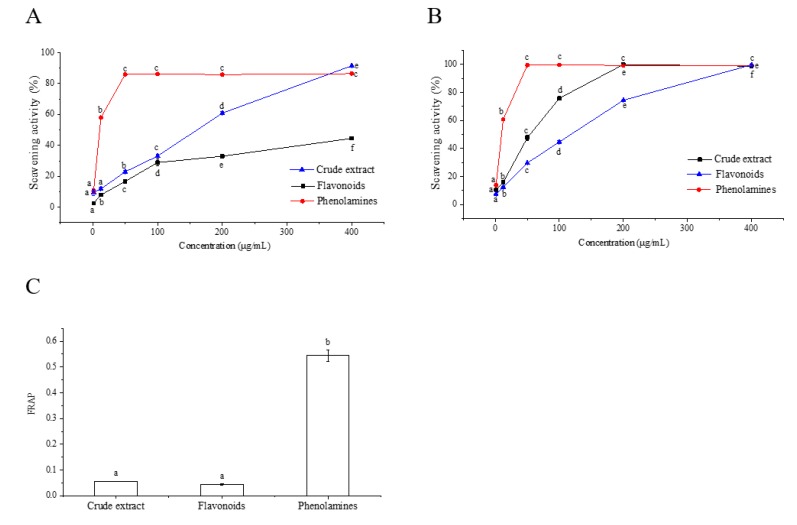
Antioxidant activity of crude extract, flavonoids and phenolamines from rape bee pollen determined by three kinds of assays. (**A**) DPPH assay, (**B**) ABTS assay, and (**C**) Ferric reducing antioxidant power (FRAP) assay.

The DPPH results are shown in Figure 3A. The DPPH radical scavenging activity varied significantly among different samples. The phenolamines showed significantly higher scavenging activity than crude extract and the flavonoids. The scavenging rate of phenolamines increased dramatically with increasing sample concentration. When the concentration reached 50 μg/mL, the DPPH radical scavenging rate of phenolamines reached above 80%, while that of crude extract and the flavonoids was below 30%. The IC50 values of crude extract and the flavonoids and phenolamines were 116.75 μg/mL, 567.945 μg/mL and 10.57 μg/mL, respectively. The ABTS radical scavenging capacity of crude extract, flavonoids and phenolamines increased in a concentration-dependent manner (Figure 3B). At the concentration of 50 μg/mL, the scavenging rate of the phenolamines reached 99.32%. The IC50 value of the phenolamines was 6.41 μg/mL, which was significantly lower than that of crude extract (76.87 μg/mL) and the flavonoids (34.19 μg/mL), indicating that the phenolamines has the strongest ABTS radical scavenging ability. A similar trend was observed in the FRAP results (Figure 3C). The phenolamines exhibited the highest ferric-ion-reducing capacity. These three assays showed that the antioxidant activity of the phenolamines was obviously higher than that of the flavonoids from rape bee pollen.

### 2.3. Cytotoxic Effects of Phenolamines and Flavonoids on HepG2 Cells

**Figure 4:**
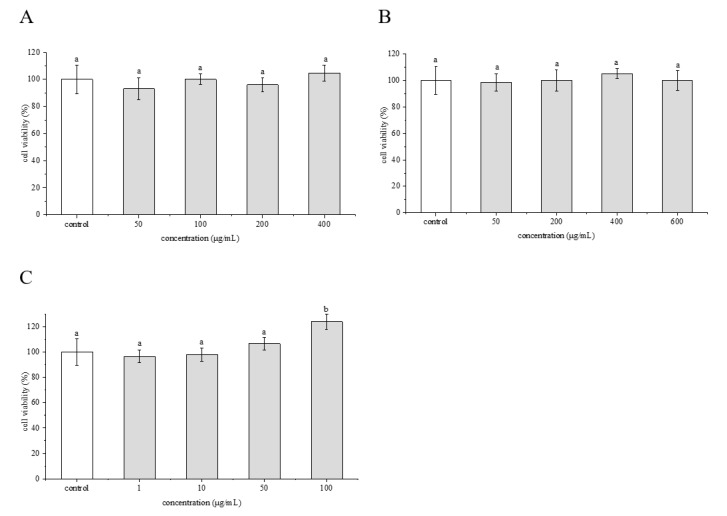
Cytotoxic effect of crude extract (**A**), flavonoids (**B**) and phenolamines (**C**) from rape bee pollen on HepG2 cells. Data are represented as mean ± SD (*n* = 6). Different letters marked above the bars indicate significant differences between groups (*p* < 0.05).

Cell viability is often used as an indicator of cytotoxicity. The cytotoxic effects of crude extract, flavonoids and phenolamines on HepG2 cells were evaluated using the MTT method (Figure 4). The crude extract at the concentrations of 50–400 μg/mL showed no obvious effect on cell viability (Figure 4A). The flavonoids at the concentrations of 50–600 μg/mL also caused no significant changes in cell survival compared with the control group (Figure 4B). The phenolamines had no effect on cell viability at concentrations below 50 μg/mL as well, whereas resulted in a significantly higher cell survival rate than the control group at concentrations above 50 μg/mL (Figure 4C). Therefore, the concentrations of <400 μg/mL for crude extract, <600 μg/mL for the flavonoids, and <50 μg/mL for the phenolamines were selected for the subsequent experiments.

### 2.4. Effect of AAPH on HepG2 Cells

**Figure 5:**
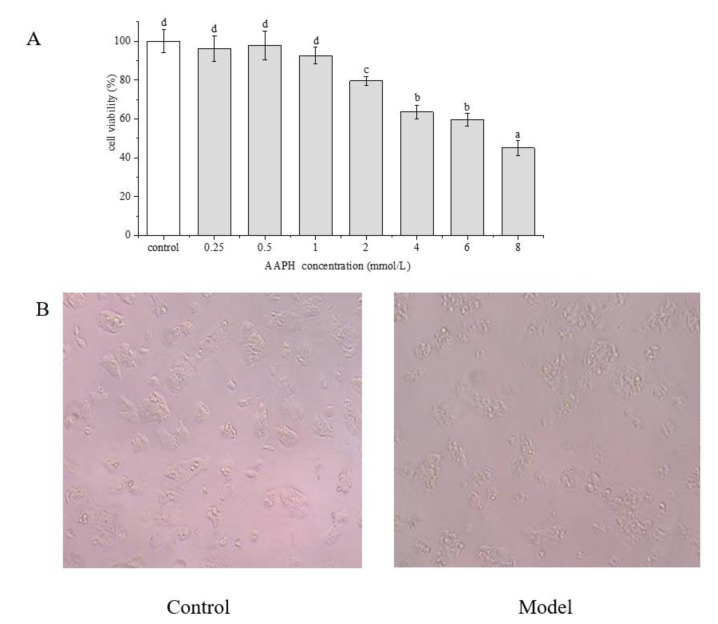
(**A**) Effect of AAPH on HepG2 cell viability. (**B**) Changes in cell morphology after the treatment of 2 mM AAPH for 24 h (model group) compared with the control group. Data are represented as mean ± SD (*n* = 6). Different letters marked above the bars indicate significant differences between groups (*p* < 0.05).

As shown in Figure 5A, the cell viability decreased in a concentration-dependent manner with an increasing concentration of AAPH. When the concentration of AAPH was ≥2 mM, the cell survival rate was significantly (*p* < 0.05) lower than that of the control group. In addition, with increasing concentration of AAPH, the cell morphology showed gradual changes. When the cells were treated with 2 mM AAPH for 24 h, the cells were significantly changed in morphology compared with the control group. The cells were contracted into irregular shapes, the intercellular space became larger, and the cell adherence became lower (Figure 5B), indicating that the cells were injured by AAPH. Therefore, 2 mM AAPH was selected for the subsequent assessments.

### 2.5. Protective Effects of Phenolamines and Flavonoids on HepG2 Cells Injured by AAPH

**Figure 6:**
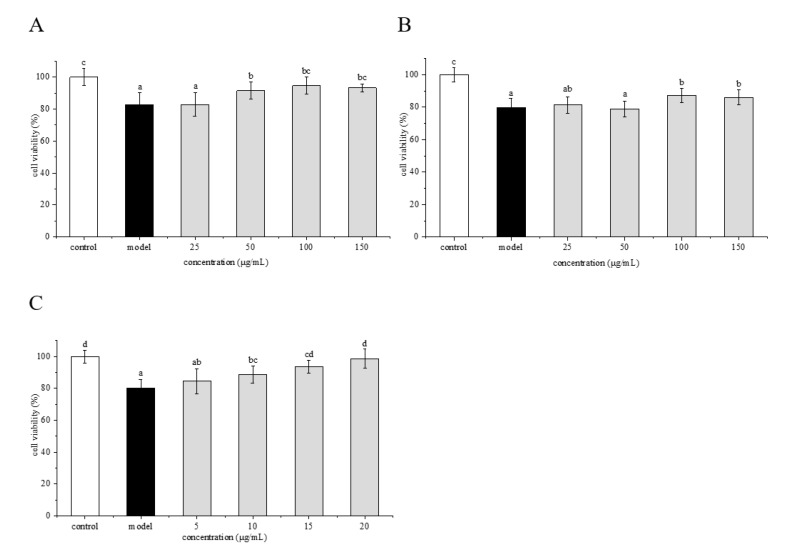
Protective effects of crude extract (**A**), flavonoids (**B**) and phenolamines (**C**) from rape bee pollen against AAPH-induced damage. Data are represented as mean ± SD (*n* = 6). Different letters marked above the bars indicate significant differences between groups (*p* < 0.05).

HepG2 cells were pretreated with crude extract, flavonoids and phenolamines, and then treated with 2 mM AAPH. As shown in Figure 6, when the cells were protected with crude extract, flavonoids and phenolamines at different concentrations, the cell survival rate increased gradually. For the crude extract treatment group (Figure 6A), the cell survival rate reached a maximum of 91.62% at the concentration of 50 μg/mL, which was significantly higher than that of the model group (*p* < 0.05), and then reached the plateau at higher concentrations. For the flavonoids treatment group (Figure 6B), the cell survival rate changed little with the increase of sample concentration at <100 μg/mL, and reached 87.14% at the concentration of 100 μg/mL, which was significantly higher than that of the model group. When the concentration was further increased to 150 μg/mL, there was no significant change in cell survival rate. For the phenolamines treatment group (Figure 6C), when the concentration was 10 μg/mL, the cell survival rate was 88.77%, which was significantly higher than that of the model group. With the increase of sample concentration, the cell survival rate increased obviously in a concentration-dependent manner. When the concentration reached 15 μg/mL, the cell survival rate was close to that of the control group, indicating the superior protective effect of phenolamines on HepG2 cells injured by AAPH. The results showed that the phenolamines have a better protective effect than crude extract and the flavonoids. Therefore, in the subsequent experiments, we focused on the mechanism of the protective effect of the phenolamines on HepG2 cells against AAPH-induced oxidative stress.

### 2.6. Preventive Effects of Phenolamines on ROS Generation

**Figure 7:**
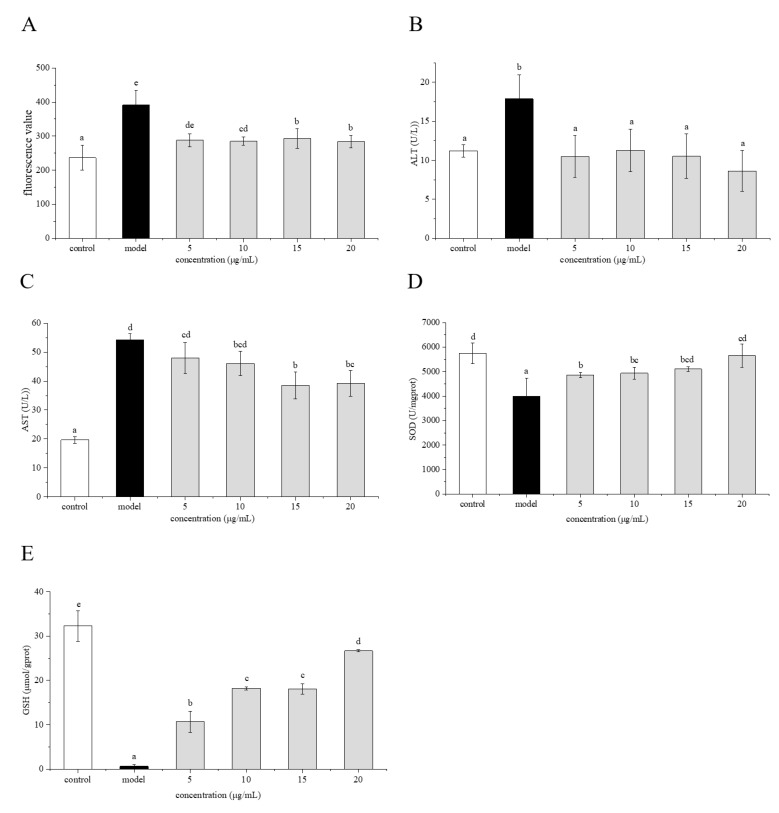
Effect of phenolamines from rape bee pollen on reactive oxygen species (ROS) (**A**), alanine aminotransferase (ALT) (**B**), aspartate aminotransferase (AST) (**C**), superoxide dismutase (SOD) (**D**), glutathione (GSH) (**E**) in HepG2 cells injured by AAPH. Data are represented as mean ± SD (*n* = 6). Different letters marked above the bars indicate significant differences between groups (*p* < 0.05).

The effect of the phenolamines on AAPH-induced ROS generation in HepG2 cells is shown in Figure 7A. The ROS level in the model group was significantly higher (*p* < 0.05) than that in the control group, indicating that AAPH led to elevated ROS levels in the cells. When the cells were pretreated with various concentrations of the phenolamines and then challenged with AAPH to induce oxidative stress, the ROS level in the cells decreased significantly (*p* < 0.05) along with increasing concentration of the phenolamines compared with that of the model group.

### 2.7. Effects of Phenolamines on AST and ALT Levels

The cells were pretreated with different concentrations of the phenolamines, followed by a challenge with AAPH. The effects of the phenolamines on aspartate aminotransferase (AST) and alanine aminotransferase (ALT) levels in HepG2 cells injured by AAPH-induced oxidative stress are shown in Figure 7B,C. The AST and ALT levels in the model group were significantly (*p* < 0.05) higher than those in the control group. When the cells were pretreated with different concentrations of phenolamines, the AST and ALT levels were obviously decreased (*p* < 0.05).

### 2.8. Effects of Phenolamines on SOD Activity and GSH Content

The effect of the phenolamines on the superoxide dismutase (SOD) activity in HepG2 cells injured by AAPH-induced oxidative stress is shown in Figure 7D. The SOD activity of cells in the control group was 5745.89 U/mgprot, while that of cells in the model group was 3999.72 U/mgprot, indicating that AAPH could reduce SOD activity in HepG2 cells. When the cells were pretreated with 5 μg/mL of the phenolamines, the SOD activity increased to 4858.79 U/mgprot, which was significantly (*p* < 0.05) higher than that of the model group. As the concentration of the phenolamines rose to 15 μg/mL, the SOD activity of the cells increased to 5102.96 U/mg protein, which is close to that of the control group. The effect of the phenolamines on glutathione (GSH) content in HepG2 cells injured by AAPH is shown in Figure 7E. The GSH content in cells of the control group was 32.25 μmol/gprot, while that of cells injured by AAPH in the model group was only 0.61 μmol/gprot. When the concentration of the phenolamines was 5 μg/mL, the GSH content was 10.68 μmol/gprot, which is significantly (*p* < 0.05) higher than that of the model group. With increasing concentration of the phenolamines, the GSH content also increased markedly (*p* < 0.05).

## 3. Discussion

Under normal physiological conditions, there is a balance between the production of ROS and the antioxidant defense mechanisms consisting of enzymes and non-enzymatic systems in human body. However, oxidative stress may occur when the production and elimination of ROS are unbalanced [26]. Oxidative stress is associated with a variety of chronic health problems such as cardiovascular disease, cancers, malaria, rheumatoid arthritis, diabetes, Alzheimer’s disease, Parkinson’s disease, and aging processes [12,13]. Therefore, high-quality antioxidants are essential to protect cells against oxidative stress injury.

Bee pollen contains abundant nutriments and bioactive components, and has been used as a health food [27]. Flavonoids and phenolamines are two important kinds of antioxidants in bee pollen. There have been many reports on the identification and biological activity of flavonoids, but fewer reports on the biological activity of phenolamines in bee pollen. Phenolamines are an important class of compounds for pollen development, viability or germination [2]. The presence of phenolamides is a distinct and persistent feature of male wild plants, and the abundance, diversity and distribution of phenolamides have been well documented [28,29,30,31,32]. Surprisingly, little is known about their biological activities. In this study, the flavonoids and phenolamines in rape bee pollen were separated and characterized, and their antioxidant activity was compared for the first time.

Phenolamine compounds are a variety of major secondary metabolites formed from the combination of phenolic compounds and polyamides. In particular, disubstituted and trisubstituted hydroxycinnamoyl amides are the major metabolites in pollen [25]. Our results showed that the phenolamines in rape bee pollen are di- and tri-substituted hydroxycinnamic acid spermidine and trisubstituted hydroxycinnamic acid spermine.

The mechanism of antioxidant capacity varies with the composition and content of antioxidants. Hence, the antioxidant capacity needs to be analyzed by different methods [33]. In this study, three assays were used to evaluate the antioxidant activity of flavonoids and phenolamines from rape bee pollen, including DPPH, ABTS and FRAP methods. The results of all three assays demonstrated that the antioxidant activity of phenolamines is much higher than that of flavonoids from rape bee pollen.

The excellent antioxidant activity of phenolamines indicates that phenolamines may have the capacity to protect cells against oxidative stress injury. Therefore, we investigated the protective effects of phenolamines against AAPH-induced oxidative stress in HepG2 cells. AAPH is a commonly used ROS radical inducer, which can continuously and stably promote ROS production in cells. Many studies have used AAPH as a free radical inducer in cell models to establish an oxidative stress model [11,14,15]. In this study, phenolamines exhibited a better protective effect than flavonoids on HepG2 cells injured by AAPH. When the cells were exposed to AAPH (2 mM), the ROS level was significantly increased, while the pre-incubation of cells with phenolamines greatly alleviated the AAPH-induced increase in ROS in a concentration-dependent manner. SOD belongs to the antioxidant enzyme system, which can counteract the formation of ROS and the toxicity of ROS to cells [13]. GSH is highly sensitive to oxidative stress and is a major antioxidant in the non-antioxidant enzyme family to help the elimination of oxidants and reduction of oxidative stress damage [34]. From our results, it can be seen that when the HepG2 cells were injured by AAPH-induced oxidative stress, the SOD and GSH levels decreased. However, pretreatment of the HepG2 cells with phenolamines significantly increased the SOD and GSH levels. Our results revealed that phenolamines have protective effects against AAPH-induced oxidative stress in HepG2 cells. The possible mechanism is that phenolamines could directly scavenge redundant free radicals and regulate intracellular antioxidant defense systems in the HepG2 cells injured by oxidative stress. The activation signaling pathway by phenolamines remains to be further studied.

## 4. Materials and Methods

### 4.1. Materials and Chemical Reagents

Rape bee pollen product was donated by Wuhan Bee Food Co., Ltd. (Wuhan, China). AB-8 resin was purchased from Nankai Hecheng Science and Technology Co. (Tianjin, China). Acetonitrile and acetic acid (HPLC grade) were obtained from J. T. Baker Co. (Phillipsburg, NJ, USA) and Aladdin Industrial Co. (Shanghai, China), respectively. We purchased 2,2-diphenyl-1-picrylhydrazyl (DPPH), 2,2’-azino-bis-3-ethylbenzothiazoline-6-sulphonic acid (ABTS), 2,2’-azobis (2-amidinopropane) dihydrochloride (AAPH) and 3-(4,5-dimethylthiazol-2-yl)-2,5-diphenylte-trazolium bromide (MTT) from Sigma (St. Louis, MO, USA). Dulbecco’s modified Eagle medium (DMEM) and fetal bovine serum (FBS) were purchased from Gibco Life Technologies (Grand Island, NY, USA). ROS assay kit with 2,7 -dichlorodihyrofluorescein-diacetate (DCFH-DA) was purchased from Beyotime Institute of Biotechnology (Shanghai, China). Total antioxidant capacity (T-AOC), aspartate aminotransferase (AST), alanine aminotransferase (ALT), superoxide dismutase (SOD) and glutathione (GSH) assay kits were obtained from Nanjing Jiancheng Bioengineering Institute (Nanjing, China).

### 4.2. Preparation of Rape Bee Pollen Crude Extract

Rape bee pollen was powdered by a pulverizer (BJ-100, Zhejiang, China). The samples were extracted by petroleum ether to remove the lipids, and subsequently extracted by 80% ethanol with ultrasound for 30 min. The ethanol extraction layer was collected, evaporated and freeze-dried to obtain the crude extract of rape bee pollen.

### 4.3. Separation of Phenolamines and Flavonoids From Crude Extract

The crude extract of rape bee pollen was subjected on a glass column (50 mm × 500 mm) wet-packed with 500 g of AB-8 macroporous resin. We dissolved 15 g of freeze-dried crude extract in 300 mL of distilled water, and loaded it on the column. The crude extract was first eluted with 4000 mL of distilled water, and then successively eluted with 4000 mL of 35% and 50% ethanol solutions, respectively. The fractions eluted with 35% and 50% ethanol solutions on the AB-8 resin column were recorded as 35% fraction and 50% fraction, respectively. The two fractions were then evaporated and freeze-dried.

### 4.4. HPLC-ESI-QTOF-MS/MS Analysis

The 35% fraction and 50% fraction were subjected to HPLC-ESI-QTOF-MS/MS analysis using an Accurate-Mass Q-TOF LC/MS 6520 (Agilent Technologies, Santa Clara, CA, USA). They were dissolved in methanol and filtered through a 0.45 µm membrane filter before injection. The separation was carried out on a Hypersil GOLD C18 column (250 × 4.6 mm, 5 µm, Thermo-Fisher, Waltham, MA, USA). The column temperature was kept at 30°C and the injection volume was 10 µL. An elution with solvent A (1% acetic acid) and solvent B (acetonitrile) in a step gradient way at a flow rate of 0.5 mL/min was carried out as follows: 0–10 min, 5–12% B; 10–15 min, 12–16% B; 15–30 min, 16–20% B; 30–40 min, 20–30% B; 40–50 min, 30–35% B; 50–60 min, 35–50% B; 60–70 min, 50–95% B; 70–90 min, 95–5% B. The detection wavelength was set at 280 nm.

ESI conditions were set as follows: negative ion mode; dry gas flow at 10 L/min, the dry gas temperature at 325 °C; nebulizer pressure at 35 psi; the capillary voltage at 3500 V; MS/MS full scan range, m/z 50–1200.

### 4.5. Antioxidant Activity

#### 4.5.1. DPPH Radical Scavenging Activity Assay

DPPH radical scavenging activity was determined by a previously described method [35] with slight modifications. DPPH solution was prepared by dissolving 8 mg of DPPH in 100 mL ethanol. 50 μL of sample was mixed with 150 μL of DPPH solution. The reaction mixture was kept for 30 min in the dark before measuring the absorbance of the mixture at 517 nm. The scavenging ability of different samples for DPPH radical was calculated by the following equation:

DPPH radical scavenging activity (%) = (1 – Asample/Acontrol)) × 100,(1)

where Asample is the absorbance of DPPH solution with sample, and Acontrol is the absorbance of DPPH solution without sample.

#### 4.5.2. ABTS Radical Scavenging Activity Assay

As previously described, this assay was carried out to determine the capacity of samples to scavenge the ABTS radical cation [8]. Stock ABTS+ solution was prepared from 7 mM of ABTS solution and 40 mM of potassium persulfate in ultrapure water. The ABTS+ solution was diluted with ethanol to obtain an absorbance of 0.700 (± 0.020) at 734 nm. ABTS+ solution of 4 mL and 0.5 mL of sample were mixed and incubated at 37 °C for 10 min. The absorbance of the mixture at 734 nm was recorded. The ABTS radical scavenging activity of the samples was calculated by the formula:

ABTS radical scavenging activity (%) = (1 – Asample/Acontrol ) × 100,(2)

where Asample is the absorbance of ABTS+ solution with sample, and Acontrol is the absorbance of ABTS+ solution without sample.

#### 4.5.3. Ferric Reducing Antioxidant Power (FRAP) Assay

The determination of FRAP was carried out according to the instructions of T-AOC kit. To carry out the experiment, 0.1 mL of samples at different concentrations were added to 3 mL of freshly prepared FRAP reagent and 300 μL of ultrapure water. After the reaction mixture was incubated at 37 °C for 10 min, the absorbance of the mixture was determined at 593 nm against a blank containing all the reagents but the sample. In the FRAP assay, the antioxidant potential of the sample was detected from a standard curve plotted using the FeSO4·7H2O linear regression equation to calculate the FRAP values of the sample.

### 4.6. Cell Culture

Human hepatic HepG2 cell lines were obtained from the Cell Bank of Institute of the Biochemistry and Cell Biology, Chinese Academy of Sciences (Shanghai, China). HepG2 cells were cultured in DMEM medium, supplemented with 10% FBS and 1% penicillin/streptomycin. All cultures were maintained in a humidified incubator with an atmosphere containing 5% CO2 at 37 °C. Cell culture medium was replaced every other day, and the cells were passaged before the cell density reached about 90%.

### 4.7. Cytotoxic Effects of Crude Extract and Different Fractions on HepG2 Cells

MTT assay was conducted to determine the cytotoxicity. HepG2 cells (1.2 × 104 cells/well) were cultured in 96-well plates. After attachment, HepG2 cells were incubated with crude extract and different fractions (35% fraction and 50% fraction) of rape bee pollen for 24 h. MTT reagent dissolved in a medium (0.5 mg/mL) was added to each well. After 4 h, the supernatant was discarded, and DMSO was added (150 μL/well). The absorbance was measured with a microplate reader (MultiScan Go, Thermo Scientific Company Ltd., Waltham, MA) at 490 nm after the cells were shaken for 10 min. The cell viability was calculated by the following equation:

cell viability (%) = [(Asample – Ablank)/(Acontrol – Ablank)] × 100,(3)

where Asample is the absorbance of cells treated with the samples, Ablank is the absorbance without cells, and Acontrol is the absorbance of untreated cells.

### 4.8. Evaluation of Viability of HepG2 Cells Treated by AAPH

Cells were seeded at 1.2 × 104 cells per well on 96-well plates and incubated in a humidified incubator containing 5% CO2 at 37 °C. After attachment, the cells were treated with AAPH at various concentrations (0–8 mM, 100 μL), and incubated for 24 h. After incubation, the cell viability was evaluated by MTT assay.

### 4.9. Assay of the Protective Effects of Crude Extract and Different Fractions on HepG2 Cells Injured by AAPH

HepG2 cells (1.2 × 104 cells/well) were cultured in 96-well plates. The cells were pretreated with various concentrations of crude extract and different fractions (35% fraction and 50% fraction) for 3 h, followed by a challenge with AAPH (2 mM). After 24 h of incubation, the cell viability was determined by MTT assay.

### 4.10. Measurement of ROS

Cellular oxidative stress owing to ROS generated by AAPH was measured using DCFH-DA-based method [13] with minor modifications. HepG2 cells were treated with different concentrations of 50% fraction (0, 5, 10, 15 and 20 μg/mL) for 24 h, incubated in DMEM with DCFH-DA solution (10 μmol/L, 100 μL) for 30 min at 37 °C, and then washed three times by phosphate-buffered saline (PBS) solution. Then, the cells were incubated with DMEM containing APPH solution for 3 h. Afterwards, the fluorescence intensity was measured using a Synergy HTX Multi-Reader (Bio-Tex, Winooski, VT) at excitation of 485 nm and emission of 530 nm. Intracellular ROS levels were expressed by longitudinal fluorescence intensity.

### 4.11. Determination of ALT and AST

The cells (3.5 × 106 cells/well) were cultured in six-well plates. After attachment, the cells were pretreated with different concentrations of 50% fraction for 3 h, and then incubated with AAPH (2 mM). After 24 h of incubation, the culture supernatant was collected, and the ALT and AST levels were measured by commercially available assay kits.

### 4.12. Measurement of SOD and GSH

The cells (3.5 × 106 cells/well) were cultured in six-well plates. After attachment, the cells were pretreated with different concentrations of 50% fraction for 3 h and then incubated with AAPH (2 mM) for 24 h. The SOD level and GSH content were then determined using the SOD and GSH assay kits, and the results were normalized to protein concentrations.

### 4.13. Statistical Analysis

Data were expressed as the mean ± standard deviation (SD). Differences among groups were evaluated by one-way analysis of variation (ANOVA) of IBM SPSS Statistics 20 for Windows (SPSS, Inc, Chicago, IL, USA) with least significant difference (Duncan) multiple range test. The value of *p* < 0.05 was considered as statistically significant.

## 5. Conclusions

In conclusion, this is the first study to separate and characterize the flavonoids and phenolamines in rape bee pollen and compare their antioxidant activity, as well as the protective effect of phenolamines against oxidative stress-induced injury. The flavonoids in rape bee pollen are mainly quercetin and kaempferol glycosides, and the phenolamines in rape bee pollen are mainly hydroxycinnamic acid spermidine and hydroxycinnamic acid spermine. The results show that phenolamines have better antioxidant activity than flavonoids. Moreover, phenolamines were found to protect HepG2 cells against oxidative stress damage induced by AAPH. Our results provide new insights into the biological activity of phenolamines in rape bee pollen.

## Figures and Tables

**Table 1:** Characterization of flavonoids in the 35% fraction of rape bee pollen by HPLC-ESI- QTOF-MS/MS in negative ion mode.

Peak	RT (min)	[M–H]^–^(*m/z*)	MS^2^ ion Fragments (*m/z*)	Proposed Formula	Error (ppm)	Tentative Identification	Reference
1	23.169	625.1402	301.0356, 463.0888	C_27_H_30_O_17_	0.86	Quercetin di-glucoside	22,23
2	23.422	609.1450	283.0253, 285.0409, 446.0856	C_27_H_30_O_16_	1.12	kaempferol di-glucoside	24
3	27.328	609.1460	255.0308, 284.0329, 285.0395, 429.0833	C_27_H_30_O_16_	0.14	kaempferol di-glucoside	24

**Table 2:** Characterization of phenolamines in the 50% fraction of rape bee pollen by HPLC-ESI-QTOF-MS/MS in negative ion mode.

Peak	RT (min)	[M–H]^–^(*m/z*)	MS^2^ ion Fragments (*m/z*)	Proposed Formula	Error (ppm)	Tentative Identification	Reference
4	28.055	436.2247	119.0500, 316.1677	C_25_H_31_N_3_O_4_	1.24	di-*p*-coumaroyl spermidine	22
5	29.079	436.2241	119.0505, 316.1673	C_25_H_31_N_3_O_4_	0.08	di-*p*-coumaroyl spermidine	22
6	29.882	436.2245	119.0498, 316.1669	C_25_H_31_N_3_O_4_	0.42	di-*p*-coumaroyl spermidine	22
7	37.234	701.3173	135.0452, 165.0560, 399.2043, 535.2560, 555.2834, 565.2671	C_38_H_46_N_4_O_9_	2.53	*p*-coumaroyl caffeoyl hydroxyferuloyl spermine	*
8	39.080	685.3245	119.0503, 145.0284, 165.0557, 399.2039, 519.2608	C_38_H_46_N_4_O_8_	0.08	di-*p*-coumaroyl hydroxyferuloyl spermine	*
9	40.087	685.3247	119.0499, 145.0287, 165.0557, 399.2045, 519.2619	C_38_H_46_N_4_O_8_	0.43	di-*p*-coumaroyl hydroxyferuloyl spermine	*
10	40.576	685.3190	119.0505, 145.0279, 165.0534, 399.2058, 519.2603	C_38_H_46_N_4_O_8_	1.68	di-*p*-coumaroyl hydroxyferuloyl spermine	*
11	45.453	439.1684	135.0447, 161.0243, 165.0556, 415.2000	C_16_H_24_N_8_O_7_	1.39	unknown	
12	46.060	439.1667	135.0448, 161.0241, 165.0553, 415.1984	C_16_H_24_N_8_O_7_	1.60	unknown	
13	47.839	582.2604	119.0491, 145.0291, 342.1464, 462.2025	C_34_H_37_N_3_O_6_	0.90	tri-*p*-coumaroyl spermidine	23
14	48.948	582.2599	119.0498, 145.0302, 342.1457, 462.2035	C_34_H_37_N_3_O_6_	1.62	tri-*p*-coumaroyl spermidine	23
15	49.033	582.2607	119.0501, 145.0296, 342.1463, 462.2027	C_34_H_37_N_3_O_6_	0.54	tri-*p*-coumaroyl spermidine	23
16	49.790	582.2602	119.0501, 145.0294, 342.1464, 462.2036	C_34_H_37_N_3_O_6_	1.28	tri-*p*-coumaroyl spermidine	23
17	50.835	582.2602	119.0494, 145.0286, 342.1459, 462.2032	C_34_H_37_N_3_O_6_	1.21	tri-*p*-coumaroyl spermidine	23

* Phenolamines identified for the first time in rape bee pollen in this study.
